# Case report: uniportal video-assisted thoracoscopic resection of a solitary fibrous tumor preoperatively predicted visceral pleura origin using dynamic chest radiography

**DOI:** 10.1186/s13019-020-01212-0

**Published:** 2020-07-08

**Authors:** Masaya Tamura, Isao Matsumoto, Daisuke Saito, Shuhei Yoshida, Munehisa Takata, Hirofumi Takemura

**Affiliations:** Department of Thoracic, Cardiovascular and General Surgery, Kanazawa University, Takara-machi 13-1, Kanazawa, 920-8640 Japan

**Keywords:** Dynamic chest radiography, Solitary fibrous tumor of the pleura, Uniportal video- assisted thoracoscopic surgery

## Abstract

**Background:**

Dynamic chest radiography (DCR) is a flat-panel detector (FPD)-based functional X-ray imaging, which is performed as an additional examination in chest radiography. DCR provides objective and quantifiable information, such as diaphragm movement, pulmonary ventilation and circulation, and is reasonable for detecting tumor invasion or adhesion.

**Case presentation:**

We present a case of Solitary Fibrous Tumor of Pleura (SFTP), preoperatively predicted visceral pleura origin using Dynamic chest radiography (DCR) and surgically resected through single-access (uniportal) video-assisted thoracoscopic surgery (UVATS).

**Conclusions:**

UVATS may be a suitable surgical option for pedunculated SFTPs. Dynamic chest radiography provides information, such as tumor invasion or adhesion and helpful for predicting origin of the tumor.

## Background

Dynamic chest radiography (DCR) is a flat-panel detector (FPD)-based functional X-ray imaging, which is performed as an additional examination in chest radiography. DCR contain a wealth of functional information, such as diaphragm movement, cardiac motion, pulmonary ventilation and circulation. The first clinical report of this technique was published by the author’s group [1]. This modality has a potential to make an accurate diagnosis of tumor invasion or adhesion to the parietal pleura, and that visual (qualitative) assessment for cancer invasion. We present a case of Solitary fibrous tumor of the pleura (SFTP), preoperatively predicted visceral pleura origin using Dynamic chest radiography (DCR) and surgically resected through single-access (uniportal) video-assisted thoracoscopic surgery (UVATS).

## Case presentation

**Fig. 1:**
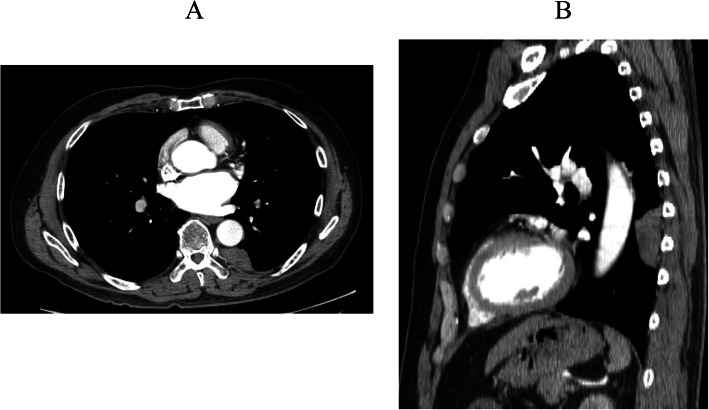
Preoperative chest computed tomography (CT) scan demonstrated a homogenous, sharply-circumscribed mass in the posterior mediastinum. **a** Enhancement showed a mass that was not enhanced. **b** Sagittal view

A 60-year-old man was admitted to our hospital for further examinations of an abnormal shadow found on a chest CT. A CT scan demonstrated a homogenous, sharply-circumscribed mass in the posterior mediastinum (Fig. 1). Additional file 1A (Video) showed preoperative DCR findings. Two points for measurements on the inspiratory frame of the dynamic-ventilation. One point was placed in the center of the targeted lesion (red point). The other point was the vertebra adjacent to the tumor (blue point). The software automatically tracked these two measuring points, and coordinates were recorded. The distance of the two points (Additional file 1B), movement in two directions (Additional file 1C) were not coincidence, which means no invasion or adhesion of the tumor to the chest wall.

**Fig. 2:**
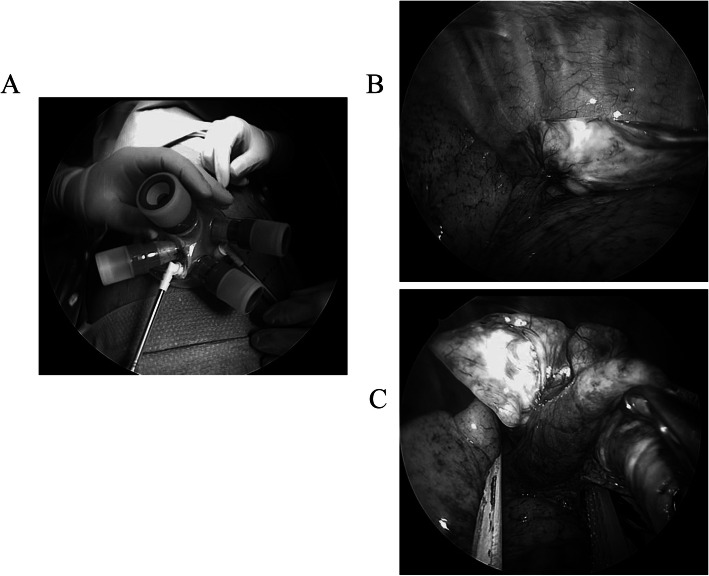
Intraoperative findings. **a** A 3.0 cm skin incision was made, and the Lap-protector (alnote LapSingleTM, Applied Alfresa Pharma Coroperation, Japan) was then placed through the incision. **b** A pedunculated tumor protruded into the thoracic cavity from the visceral pleura, and was attached to the left lower lobe. **c** The tumor stalk was resected using an articulating endostapler (Covidien, Norwalk, CT, USA)

The patient had a CT guided biopsy, and histopathology examination revealed benign solitary fibrous tumor. However, surgery was required for curative resection. The patient was administered under general anesthesia using one-lung ventilation and was placed in a lateral position. The 3 cm incision located of the midaxillary line at the level of the sixth intercostal space, and the Lap-protector (alnote LapSingleTM, Applied Alfresa Pharma Coroperation, Japan) was then placed through the incision (Fig. 2a). We applied CO2 gas insufflation to push the lung down. We start the CO2 insufflation at 3 l/min, with a pressure of 5 mmHg, while the CO2 still allows for a generous space which allows the surgery to be undertaken. A pedunculated tumor that protruded into the thoracic cavity from the visceral pleura was observed. The tumor was attached by its stalk to the left lower lobe, and moved freely (Fig. 2b). We believed that a single-port surgery was feasible for this type of lesion. The tumor stalk was resected using an articulating endostapler (Covidien) (Fig. 2c).

**Fig. 3:**
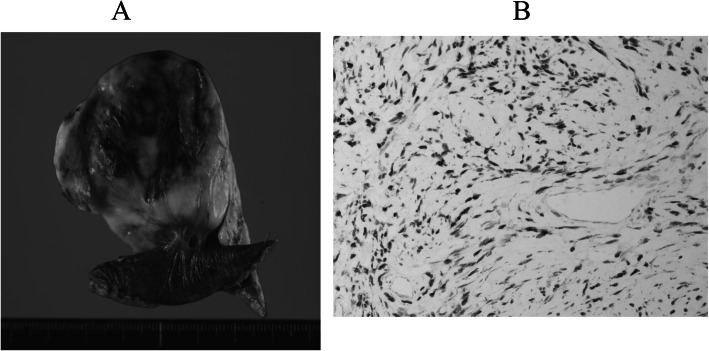
**a** Cut surface of the resected specimen demonstrated a smooth margin with solid consistency and a heterogeneous appearance (7.5 × 4.7 × 1.5 cm). **b** Histological specimen demonstrated that the tumor consisted of hypocellular and moderately cellular areas of bland spindle cells in abundant collagen fibers. (Hematoxylin and eosin, × 200)

Microcopically, the tumor was characterized by spindle cells that were organized in short fascicles against a collagenous background. Areas of high cellularity and mitotic activity were absent (Fig. 3). Immunohistochemical staining was strongly positive for CD34, STAT6 in the cytoplasm of the tumor cells. The final pathology was determined a benign SFTP. The patient was followed-up for 6 months, and no evidence of recurrence was observed.

## Discussion

Dynamic chest radiography provides objective and quantifiable information, such as diaphragm movement, pulmonary ventilation and circulation, and is reasonable for detecting tumor invasion or adhesion. Sakuma et al. [2] reported that dynamic-ventilation CT can be utilized as a novel imaging approach for the preoperative assessment of pleural invasion and adhesion. The total radiation exposure for the dynamic ventilation CT ranged from 4.2 to 6.1 mSv. The total patient dose of DCR is 0.23 mSv, about double that of conventional chest radiography [1]. The DCR is acceptable because of the increased yield of information, low radiation exposure, simple and rapid means of functional imaging. Since chest X-ray is routinely performed as a standard preoperative assessment of patients with lung cancer, we currently believe that the addition of DCR to conventional chest X-ray is reasonable for obtaining diagnosis of tumor invasion or adhesion of the parietal pleura. A prospective study enrolling a larger number of patients is now ongoing.

SFTP is a relatively rare pleural tumor that generally arises from the visceral pleura as an asymptomatic pleural-based mass [3]. Although most SFTPs are considered as histologically benign, local recurrences and enlargements without any signs of invasion or metastasis have been reported [4]. SFTP usually arises from the visceral pleura, although an origin from the parietal or the diaphragmatic pleura has been reported in about 20% of the cases [5].

Recently, VATS has become a more commonly used technique for thoracic tumor surgeries [6]. In particular, UVATS has been useful for specific diseases, such as pneumothorax [7]. Pulmonary anatomical resections through UVATS started in 2010 [8], and then have experienced a huge worldwide spread. Nowadays, there are reports of uniportal tracheal resection and reconstruction, bronchoplastic procedures, lobectomies with en bloc chest wall excision, and vascular reconstruction with optimal outcomes [9]. Because only one intercostal space is involved, the possible advantages of UVATS include less postoperative pain, fewer postoperative drainage days, shorter hospital stays, and cosmetic advantages compared with those of conventional three-port VATS. Some authors have reported less postoperative pain and less paresthesia in patients who underwent minor procedures through a single-port approach compared with the classical three-port approach [10, 11].

There are obvious technical problems with UVATS. UVATS is not a naturally ergonomic procedure, because the traditional thoracoscopic principles of triangulation are lost. In addition, positioning of multiple devices poses a problem because they are passed through a single small incision in the chest. We applied CO2 gas insufflation to push the lung and diaphragm down using uniportal thoracoscopic surgery port. The CO2 insufflation is known to make the uniportal VATS procedure easier by flattening the lung and diaphragm.

## Conclusion

We recommend the minimally invasive UVATS for resecting a thoracic pedunculated SFTP. DCR may play a great role for predicting the origin of the tumor preoperatively.

## Supplementary information

**Additional file 1.** Dynamic chest radiography findings. A: Video showed preoperative DCR findings. B: Red point; the center of the targeted lesion. Blue point; the vertebra adjacent to the tumor. The Figure shows the distance of the two points. C: Movement in two directions were not coincidence, which means no invasion or adhesion of the tumor to the chest wall.

## Data Availability

Not applicable.

## Abbreviations

DCR: Dynamic chest radiographys
SFTP: Solitary fibrous tumor of the pleura
UVATS: Uniportal video-assisted thoracoscopic surgery
